# A quantitative genetic approach to assess the evolutionary potential of a coastal marine fish to ocean acidification

**DOI:** 10.1111/eva.12248

**Published:** 2015-02-13

**Authors:** Alex J Malvezzi, Christopher S Murray, Kevin A Feldheim, Joseph D DiBattista, Dany Garant, Christopher J Gobler, Demian D Chapman, Hannes Baumann

**Affiliations:** 1 School of Marine and Atmospheric Sciences, Stony Brook UniversityStony Brook, NY, USA; 2 Department of Marine Sciences, University of ConnecticutGroton, CT, USA; 3 Pritzker Laboratory for Molecular Systematics and Evolution, Field Museum of Natural HistoryChicago, IL, USA; 4 Red Sea Research Center, King Abdullah University of Science and TechnologyThuwal, Saudi Arabia; 5 Département de Biologie, Université de SherbrookeSherbrooke, QC, Canada

**Keywords:** animal model, ASReml, Atlantic Silverside *Menidia menidia*, genotyping, heritability, microsatellites, pedigree analysis, survival

## Abstract

Assessing the potential of marine organisms to adapt genetically to increasing oceanic CO_2_ levels requires proxies such as heritability of fitness-related traits under ocean acidification (OA). We applied a quantitative genetic method to derive the first heritability estimate of survival under elevated CO_2_ conditions in a metazoan. Specifically, we reared offspring, selected from a wild coastal fish population (Atlantic silverside, *Menidia menidia*), at high CO_2_ conditions (∼2300 μatm) from fertilization to 15 days posthatch, which significantly reduced survival compared to controls. Perished and surviving offspring were quantitatively sampled and genotyped along with their parents, using eight polymorphic microsatellite loci, to reconstruct a parent–offspring pedigree and estimate variance components. Genetically related individuals were phenotypically more similar (i.e., survived similarly long at elevated CO_2_ conditions) than unrelated individuals, which translated into a significantly nonzero heritability (0.20 ± 0.07). The contribution of maternal effects was surprisingly small (0.05 ± 0.04) and nonsignificant. Survival among replicates was positively correlated with genetic diversity, particularly with observed heterozygosity. We conclude that early life survival of *M. menidia* under high CO_2_ levels has a significant additive genetic component that could elicit an evolutionary response to OA, depending on the strength and direction of future selection.

## Introduction

Ocean acidification (OA) has been recognized among the key anthropogenic processes threatening humanity and its environment (Rockstrom et al. 2009). Concerns about OA stem from (i) the certainty of continued oceanic CO_2_ uptake with projected further atmospheric CO_2_ increases and hence further declines in ocean pH and calcium carbonate saturation (Caldeira and Wickett 2003; Sabine et al. 2004; Bates et al. 2012) and (ii) from fast accumulating evidence that such predicted conditions affect many contemporary marine organisms in complex and often negative ways (Hendriks et al. 2010; Doney et al. 2012). High OA sensitivities, as inferred from reductions in survival, growth, and calcification rates under experimentally elevated CO_2_ conditions, have been observed during the early life stages of calcifying marine invertebrates (Orr et al. 2005; Kleypas et al. 2006; Doney et al. 2009; Talmage and Gobler 2010), but also in offspring of some marine fishes. In the latter, documented adverse effects of high CO_2_ exposure range from behavioral abnormalities (Munday et al. 2009, 2014; Dixson et al. 2010, 2014; Chivers et al. 2014) and tissue damage (Frommel et al. 2012b) to directly reduced growth and survival rates (Baumann et al. 2012; Chambers et al. 2014). Cumulatively, these findings imply that OA could profoundly alter marine ecosystems, likely to the detriment of humans (Branch et al. 2013; Bednaršek et al. 2014).

Detecting widespread CO_2_ sensitivities in marine organisms, however, is only a first step in assessing OA's long-term ecological consequences (Pfister et al. 2014; Sunday et al. 2014). Although anthropogenic OA is likely unprecedented in both pace and magnitude (Caldeira and Wickett 2003), the predicted environmental shifts will still happen gradually over the next few hundred years, that is, within a few (e.g., whales, turtles, many sharks) or hundreds (e.g., many fish, mollusks) or hundred thousands of generations (single cell plankton). Hence, an equally important but much less understood question is to what extent organisms will adapt to acidifying oceans through natural selection of either extant or randomly arising genotypes of greater fitness. This knowledge gap is being increasingly recognized, and several approaches have so far been used to address it (Munday et al. 2013; Reusch 2013; Sunday et al. 2014).

One approach has been to demonstrate standing genetic variation in CO_2_ reaction norms within marine species or populations. For example, different strains of cyanobacteria from different oceanic regions show large variations in CO_2_-dependent nitrogen fixation rates, suggesting that some strains benefit from increasing CO_2_ more than others (Hutchins et al. 2013). Differentially adverse CO_2_ effects are also evident, for example, between strains of the coccolithophore *Emiliania huxleyi* (Langer et al. 2009), or genetically different oyster *Saccostrea glomerata* lines (Parker et al. 2011), sea urchin populations (Kelly et al. 2013), and Atlantic cod *Gadus morhua* populations (coastal Norway versus Baltic Sea, Frommel et al. 2012a,b). Such observations suggest that rising CO_2_ levels are gradually shifting the fitness landscape in the ocean, thus triggering changes in the genotypic composition of marine species, even in cases where little or no phenotypic change will be observed (Pespeni et al. 2013; Sunday et al. 2014).

A second type of approach is to assess evolutionary responses to OA *in vitro*. Lohbeck et al. (2012) found that adverse growth and calcification effects of high CO_2_ (1000 μatm) in single clones of *E. huxleyi* partially disappeared after rearing 500 asexual generations at high CO_2_ levels in the laboratory, suggesting a *de novo* evolutionary response. Unfortunately, such *in vitro* approaches are largely unfeasible in metazoans with substantially longer generation times. *In vitro* selection for OA-relevant genes was demonstrated via analysis of single nucleotide polymorphisms (SNPs) in purple sea urchin *Strongylocentrotus purpuratus* larvae cultured under different CO_2_ levels (Pespeni et al. 2013). This revealed consistent shifts in allele frequencies predominantly within functional genomic groups associated with calcification and osmoregulatory control.

A third type of approach has been to infer the evolutionary potential of a trait by estimating its heritability *h*^2^ (Charmantier and Garant 2005; Garant and Kruuk 2005; Sunday et al. 2014). Heritability is the proportion of a traits interindividual phenotypic variation that is not environmentally but genetically determined. Knowing a traits heritability is valuable, because together with the strength of selection (selection coefficient, singe-trait *S*, multiple traits *β*), the rate of evolutionary change (*ΔZ*) can be estimated with the breeders equation (Δ*Z* = h^2^S, Falconer and Mackay 1996). Thereby, highly heritable traits that come under strong selection will evolve rapidly within few generations, whereas low heritability combined with small selection differentials produce small or negligible evolutionary change (but see Merilä et al. 2001; Morrissey et al. 2010).

To experimentally estimate a traits heritability, mean phenotypic change (*ΔZ*) could be measured following selection over multiple generations, but as with experimental evolution, this is practical mostly for organisms with short generation times (days to weeks, Lohbeck et al. 2012). Alternatively, trait heritability can be quantified in single-generation experiments, provided the design allows the different components of phenotypic variance (*V*_P_) to be distinguished (Lynch and Walsh 1998; Sunday et al. 2014). This can be achieved by measuring *V*_P_ in offspring of single mother × father (=dam × sire) crosses that are separately reared under standardized laboratory conditions. Unfortunately, this can necessitate an impractically large number of experimental vessels (e.g., Sunday et al. 2011: >1000), particularly if model species or later developmental stages demand larger rearing volumes (e.g., fish larvae) or if the genetic diversity of a wild population is to be represented (20+ spawners per sex, Conover and Present 1990; Baumann and Conover 2011a). Finally, heritability estimates associated with OA are most needed for life history traits (survival, growth, fecundity), which are most directly related to fitness (Mousseau and Roff 1987; Sunday et al. 2014).

Here we evaluate a quantitative genetic approach to estimate trait heritability during single-generation experiments that involve large numbers of spawners but without the onerous requirement of separate experimental units for each sire × dam combination. Instead, the approach relies on resolving genetic relationships between offspring and parents *post-mortem* by genotyping all individuals using microsatellite markers. Microsatellites are routinely used to assign parentage with high confidence, for example, in human paternity tests (Marshall et al. 1998) or studies of wild population genetics (Chapman et al. 2009; Feldheim et al. 2014). Here, the approach is applied to derive the first heritability estimate for larval survival under elevated CO_2_ conditions in a marine fish. We chose the Atlantic silverside (*Menidia menidia*), because (i) it is an ecologically important, annual forage fish along most of the western Atlantic coast, (ii) elevated CO_2_ levels have previously been shown to reduce early life survival in this and related species (Baumann et al. 2012; Murray et al. 2014), and (iii) the required species-specific primers for 10 microsatellite loci were already available (Sbrocco and Barber 2011).

To explore the approach, we reared newly fertilized offspring from wild-caught parents at CO_2_ conditions projected for the average open ocean within the next 300 years (∼2000 μatm, Caldeira and Wickett 2003), while quantitatively sampling all perished and surviving larvae for downstream genetic analyses. Thus, we determined ‘*D*ays *S*urvived at high CO_2_’ (hereafter: *DS*) and the genotype of each larva, which were used to construct and analyze a parent–offspring pedigree. We then tested the null hypothesis that genetically related offspring were no more phenotypically similar (i.e., survived similarly long) than unrelated offspring. Other genetic parameters such as observed heterozygosity or allelic richness were quantified for each replicate to examine the relationship between genetic diversity and offspring survival in high CO_2_ environments.

## Materials and methods

### Parental collection and laboratory rearing

We collected ripe, adult *M. menidia* at the beginning of the spawning season (25 April 2013) from a tidal salt marsh on the North shore of Long Island (Poquot, 40°58.12′N, 73°5.28′W). Males and females were caught with a 30 × 2 m beach seine, then transported to our laboratory facility (Flax Pond Marine Laboratory) and held overnight in separate temperature-controlled baths (200 L, 21°C). The next morning, 29 females were strip-spawned by gently squeezing their hydrated eggs into a large shallow container containing seawater-activated sperm of 42 males and a large sheet of window screen (1 mm mesh). Within 15 min, fertilized eggs attached to the screen via uncoiled chorionic filaments, in contrast to unfertilized eggs that could later be gently rinsed off. The window screen was randomly cut into many small pieces, and within 2 h after fertilization, 3–6 randomly selected pieces totaling 100 embryos were suspended into each of 10 replicate rearing containers (20 L) preset for the high CO_2_ treatment (∼2300 μatm, Table1). Another 100 embryos were placed into each of five control replicates (∼460 μatm). The high CO_2_ treatment represents the maximum level predicted for the open ocean within the next 300 years (Caldeira and Wickett 2003), but also a seasonal condition occurring within many productive coastal habitats already today (Wallace et al. 2014). Control replicates were used to validate the assumption that high CO_2_ conditions indeed reduced offspring survival, as has been documented in previous studies on this and closely related species (Baumann et al. 2012; Murray et al. 2014). Throughout the experiment, temperature, salinity, and photoperiod were held constant at 24°C, 25 psu, and 15L:9D, respectively. Larvae hatched ∼120 h postfertilization and were immediately provided with daily *ad libitum* rations of newly hatched brine shrimp nauplii, *Artemia salina*, (San Francisco strain; Brine Shrimp Direct, Inc. Ogden, UT, USA) until the end of the experiment.

**Table 1:** Carbon chemistry parameters during the experiment as determined from discrete water samples

Treatment	Replicates	pH (NIST)	pCO_2_	Total dissolved inorganic carbon	Total alkalinity
Control	5	8.11 ± 0.07	459 ± 73	2175 ± 126	2262 ± 123
High CO_2_	10	7.45 ± 0.04	2294 ± 134	2324 ± 166	2235 ± 179

### Seawater chemistry manipulation

Seawater chemistry was modified and monitored throughout the experiment according to recommended best practices for OA research (Riebesell et al. 2010). We used a gas proportioning system (Cole Parmer® flowmeters on a multitube frame, Cole Parmer, Vernon Hills, IL, USA) to deliver precise mixes of 5% ultra pure CO_2_ and air (air only for controls) directly to the bottom of each rearing container via air stones. Target pH for high CO_2_ (∼2300 μatm, pH = 7.45) and control levels (∼460 μatm, pH = 8.10) were monitored daily with an Orion ROSS Ultra pH/ATC Triode and Orion Star A121 pH portable meter, which were calibrated regularly using three-point National Institute of Standards and Technology (NIST) traceable pH references. In addition, discrete water samples were taken once from each replicate/treatment (borosilicate bottles, preserved with 200 μL HgCl_2_) and analyzed for dissolved inorganic carbon (DIC, mol kg seawater^−1^) with an EGM-4 Environmental Gas Analyzer (for detailed descriptions see Talmage and Gobler 2010; Gobler et al. 2014). Actual levels of CO_2_ and total alkalinity were then calculated based on measured DIC, pH (NIST), temperature, salinity, and first and second dissociation constants of carbonic acid in seawater (Roy et al. 1993) using CO2SYS (http://cdiac.ornl.gov/ftp/co2sys/, Table1).

### Estimation of ‘Days survived at high CO_2_’ (DS)

**Figure 1:**
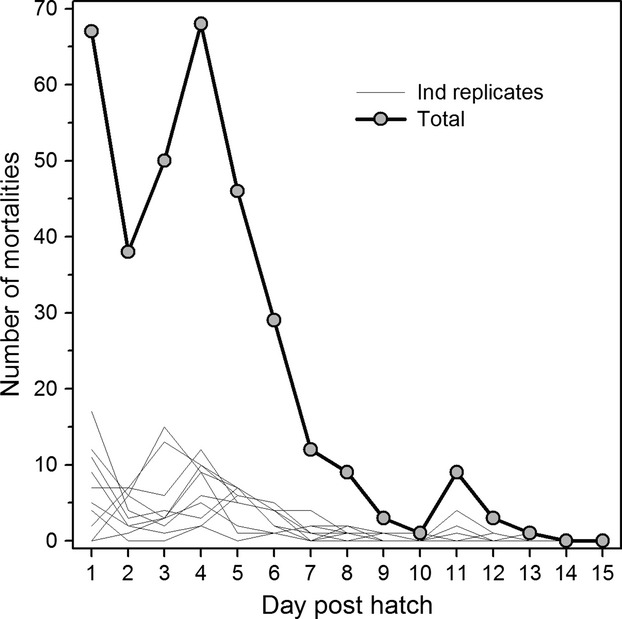
*Menidia menidia*. Daily posthatch mortality at high pCO_2_ levels (2300 μatm) in each of 10 replicates (thin gray lines). The bold, black line depicts the total mortality across all replicates.

To quantify *DS* for each hatched larva, the bottom of every high CO_2_ rearing container was gently siphoned twice daily, and all dead individuals were removed, recorded, and individually transferred to a tissue lysis solution (100 μL tissue lysis buffer + 12 μL proteinase K) for subsequent genomic DNA extraction. Frequent siphoning was critical, because fish larvae decompose beyond recognition within hours after death (at 24°C). The pattern of daily posthatch mortality was typical for early larval fish, with mortality peaking soon after hatch (1–4 dph), but then declining exponentially over the remaining days of the experiment (Fig.1). After two consecutive days without any mortality (days 14 and 15 posthatch), the experiment was terminated and all surviving larvae (i.e., *DS* = 15) were individually transferred to tissue lysis solution for DNA extraction. To specifically evaluate the resistance of the population to elevated CO_2_, only hatched larvae of the 10 high CO_2_ replicates were sampled for genetic analyses. Unhatched embryos were recorded but not genotyped. To validate the assumption that high CO_2_ was a significant source of mortality in the experimental replicates, live larvae were counted on day 10 posthatch by gently scooping small groups into replacement containers.

### DNA extraction and amplification

Adult spawner DNA was extracted from tail clips (15–35 mg) following the animal tissue protocol of Qiagen DNeasy® kits. Larval DNA (*DS* ranging from 1 to 15 dph) was extracted using a more cost-effective ‘salting out’ protocol (Sunnucks et al. 1996) that yielded useable DNA from larval silversides. Ten polymorphic microsatellite loci for *M. menidia* (Sbrocco and Barber 2011) were amplified for all parents and offspring in a 10 μL reaction containing 1× PCR buffer, 10× bovine serum albumin, 1.5–3.5 mm MgCl_2_ (Table2), 0.12 mm dNTPs, 0.16 μm of the reverse primer and fluorescently labeled M13 primer (Schuelke 2000), 0.04 μm of the species-specific forward primer and 1 unit Taq polymerase, and 10–40 ng of genomic DNA. Thermal cycling consisted of 5 min at 94°C followed by 35 cycles of 94°C for 30 s, primer specific annealing temperature (*T*_a_, Table2) for 30 s and 72°C for 60 s with a final extension at 72°C for 10 min. Fluorescently labeled PCR products were electrophoresed on an ABI 3730 DNA analyzer along with an internal fluorescent ladder (LIZ-500; Applied Biosystems). Alleles were scored by a single analyst (AJM) using the software Peakscanner 1.0 (Applied Biosystems®, Life Technologies, Grand Island, NY, USA). A subset of approximately 10% of genotypes was verified by a second analyst (KAF) using GENEMAPPER v4.0 (Applied Biosystems). One locus (Mm09) did not amplify and another one did not yield easily scored peaks (Mm119), hence only 8 of 10 loci were eventually used for parentage assignment and genetic analyses.

**Table 2:** Allele frequency summary statistics for eight microsatellite loci analyzed for all parent and offspring *Menidia menidia*: number of alleles (*K*), annealing temperature (*T*_a_), salt concentration used (MgCl_2_), number of individuals typed (*N*), observed heterozygosity (*H*_Obs_), expected heterozygosity (*H*_Exp_), and polymorphic information content (PIC)

Locus	*T*_a_	MgCl_2_	*K*	*N*	*H*_Obs_	*H*_Exp_	PIC
002	56	2.5	18	636	0.700	0.898	0.889
108	47	1.5	8	800	0.668	0.755	0.716
202	45	3.5	12	759	0.768	0.783	0.753
204	42	3.5	16	807	0.664	0.850	0.833
240	42	2	12	789	0.791	0.811	0.787
248	51	1.5	10	718	0.758	0.787	0.752
251	47	3	21	757	0.682	0.858	0.844
272	44	3.5	19	802	0.643	0.86	0.845

### Genetic analyses

We used CERVUS 3.0 (www.fieldgenetics.com) to calculate summary statistics (e.g., number of occurrences of each allele at each locus) and assess the suitability of loci for parentage analysis (Table2). CERVUS was then used to calculate the following four measures for each of the 10 high CO_2_ replicates: relative allelic richness (i.e., N_Alleles, replicate_/N_Alleles, total_), observed heterozygosity, number of dams, and number of sires. Linear regression was used to relate the four proxies to survival. Subsequently, CERVUS was used to assign individual offspring to candidate mothers and fathers. For each offspring tested, candidate parents were assigned with at least 95% confidence using exclusion probability first and the maximum likelihood score second (Marshall et al. 1998). Comparing each offspring to all parent genotypes, exclusion of candidate parents followed if mismatches occurred at more than one locus. In total, 704 of 772 individuals (91%) were successfully assigned to parents, a success rate comparable to other studies on fish (75–95%; Estoup et al. 1998; Eldridge et al. 2002; Vandeputte et al. 2004). Unassigned offspring, resulting from poor amplification at some loci, were excluded from further analyses. All females, but only 35 of 42 males, were assigned to offspring, likely because the sperm from seven males was not activated or the fertilized eggs were not selected for in the experiment.

Last, we ran a hierarchical set of univariate ‘animal models’ for the trait *DS*, using the restricted maximum likelihood (REML) software ASReml v3.0.5 (©VSN International Ltd, Wood Lane, Hemel Hempstead, UK). An ‘animal model’ is a mixed model (i.e., a form of linear regression with ‘fixed’ and ‘random’ effects as explanatory variables) that has been found advantageous for estimating phenotypic variance components in wild populations (Lynch and Walsh 1998; Kruuk 2004; Garant and Kruuk 2005; Thériault et al. 2007; Dibattista et al. 2009). For fixed effects, we only considered replicate *ID* (1–10), which corresponds to the experimental vessel, given that age (all individuals had the same day of fertilization) and sex (individuals sampled in their entirety) were not relevant factors. Replicate *ID* significantly influenced *DS* (df = 9, *P* < 0.001, univariate general linear model SPSS; IBM) therefore all subsequent analyses included replicate *ID* as a fixed effect to remove its effect prior to the estimation of genetic parameters. We then estimated the heritability of *DS* by testing three distinct models: (i) the base model including only the fixed effect, (ii) a model including the fixed effect and an additive genetic random effect (*α*_*i*_ with variance *V*_A_), and (iii) a model including the fixed effect, additive genetic random effect, and dam identity (*m*_i_ with variance *V*_M_) as a random effect. The components of total phenotypic variance in *DS* (*V*_DS_ = *V*_A_ + *V*_M_ + *V*_R_) were then used to calculate narrow sense heritability *h*^2^ of *DS* as the ratio of additive genetic variance to total phenotypic variance (*h*^2^ = *V*_A_/*V*_DS_) and maternal effects *m*^2^ of *DS* as the ratio of maternal variance to total phenotypic variance (*m*^2^ = *V*_M_/*V*_DS_). Statistical significance was assessed with likelihood-ratio tests [LRT = −2 * (likelihood base model–likelihood model with random effects)] tested against the chi-square distribution (df = 1).

## Results

**Figure 2:**
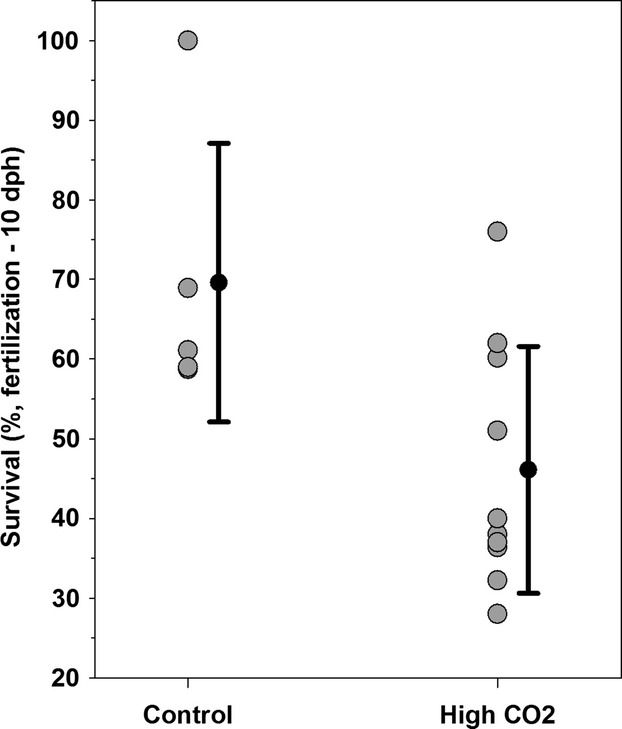
*Menidia menidia*. Survival from fertilization to 10 dph in offspring exposed to high (2300 μatm) versus ambient (control: 460 μatm) pCO_2_ levels.

High CO_2_ conditions in the experimental replicates significantly lowered average offspring survival by 34% compared to control replicates (10 dph survival ± SD: High CO_2_ = 46.1 ± 15.5%, Control: 69.6 ± 17.5%, *t*-test, df = 13, *t* = 2.63, *P* = 0.02, Fig.2). In the experimental replicates, 843 of 1000 fertilized embryos hatched, and of these 336 dead and 436 surviving individuals were recovered throughout the experiment. The remaining 7.1% were unaccounted for, likely due to rapid decomposition between siphoning. We assumed that decomposition rates were independent of genotype and therefore did not bias our results.

**Figure 3:**
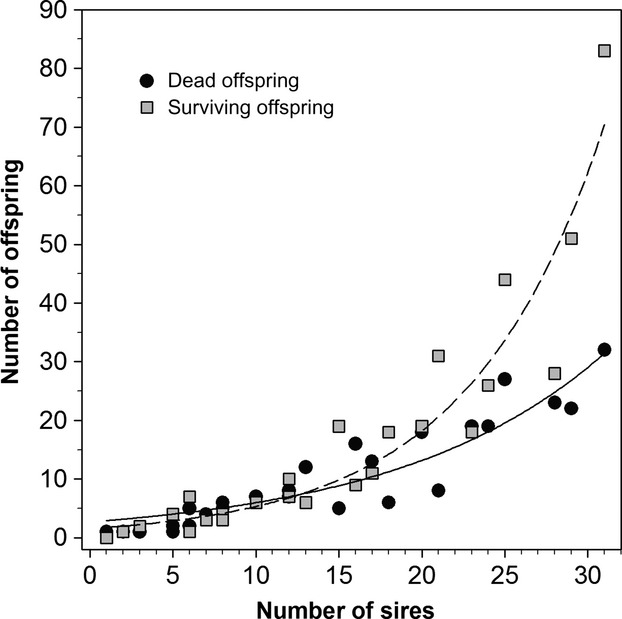
*Menidia menidia*. Relationship between the number of offspring from each female and the number of sires detected to fertilize offspring from each female. Black circles denote offspring that perished during the experiment, whereas gray squares depict survivors (i.e., the data are paired, such that each female is represented once along the *x*-axis). Both perished and surviving offspring were fitted with an exponential curve (dashed and solid line, respectively). *N*_Dead_ = 2.7 × e^0.08 × Sires^, *R*^2^ = 0.85, *P *< 0.0001; *N*_Surv_ = 1.56 × e^0.12 × Sires^, *R*^2^ = 0.89, *P *< 0.0001.

**Figure 4:**
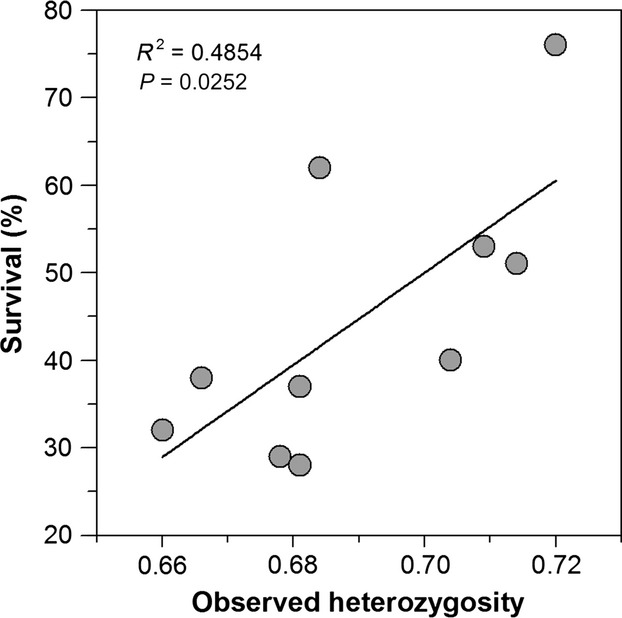
*Menidia menidia*. 15 dph offspring survival at high CO_2_ conditions in relation to observed heterozygosity at 10 replicate rearing containers.

Pedigree analysis revealed that the minimum number of sires per dam was one, resulting in one offspring (dead on day 7) produced from that pair. The maximum number of sires to fertilize a single dam's eggs was 31, resulting in 115 offspring, of which 83 survived and 32 died (Fig.3). Dams with the most offspring were also sired by the most males, and this increase in offspring number with sire number was exponential and greater for survivors than for perished offspring (Fig.3). Survival among the 10 high CO_2_ replicates ranged from 28% to 76%, with a mean (SD) survival of 46% (15%). Survival tended to increase with all four measures of genetic diversity (relative allelic richness, observed heterozygosity, number of dams, number of sires); however, the linear relationship was only significant for observed heterozygosity (*P* = 0.025, Fig.4).

The estimated genetic variance components for *DS* are presented in Table3. Including genotype as an additive genetic random effect to the base model improved the model significantly (LRT = 22.26, *P* < 0.001). Adding maternal identity as a second random effect resulted in a small and nonsignificant model improvement (LRT = 2.20, *P* = 0.14). Hence, in the final model without maternal identity, the estimated total phenotypic variance (±SE) *V*_DS_ was 29.15 ± 1.69, with an additive genetic variance *V*_A_ of 5.72 ± 2.10 and thus a heritability of larval survival at high CO_2_ of 0.20 ± 0.07.

**Table 3:** Variance component estimates for two univariate animal models predicting ‘days survived at high CO_2_’ (DS) as a function of replicate ID (fixed effect) and genotype *α*_*i*_

Model	Variance component	Estimate	Standard error
*DS*_*i*_ = *ID* + *α*_*i*_ + *ε*_*i*_	*V*_DS_	29.145	1.685
	*V*_A_	5.716	2.102
	*V*_R_	23.428	1.925
Heritability	*h*^2^ = *V*_A_/*V*_DS_	0.196	0.067

*V*_DS_, total phenotypic variance of *DS*; *V*_A_, additive genetic variance (genotype); *V*_R_, residual variance.

## Discussion

We used a quantitative genetic approach to estimate the heritability of fish survival during the early developmental stages at elevated CO_2_ conditions. Our approach had three priorities, that is, using a (i) single-generation experiment with (ii) enough parents to approximate a wild population's genetic diversity, while (iii) allowing all sire × dam crosses to be reared in a (replicated) common garden environment. We found that genetically related larvae were indeed phenotypically more similar (i.e., survived similarly long at elevated CO_2_ conditions) than unrelated larvae, which translated into a significant component of additive genetic variance and a small but nonzero heritability of 0.20 ± 0.07. Compared to average larval survival at the controls (70%), elevated CO_2_ conditions significantly reduced survival (to 46%, a 34% reduction) in addition to CO_2_-unrelated mortality agents. Hence, our heritability estimate for survival under elevated CO_2_ conditions should be considered a maximum, because it assumed that all mortality resulted from these conditions.

The small contribution of maternal effects was unexpected, given that early life history traits in marine organisms often reflect maternal traits and investment (Green 2008; Sunday et al. 2011; Gao and Munch 2013). A low likelihood of detecting maternal effects in this case might have been due to collecting all adults from the same site and rearing offspring in common garden environments at unrestricted food levels (Marshall and Uller 2007; Allan et al. 2008). We also detected a positive relationship between observed heterozygosity and early life survival at high CO_2_ levels. Assuming that neutral genetic variation is a proxy for adaptive genetic variation, this finding highlights the importance of standing genetic variation for a population's capacity to withstand environmental stress.

When evaluating the usefulness of our approach, its advantages (i.e., single-generation, genetic representation, common garden rearing) should be weighed against feasibility, cost, and time considerations. In most OA-sensitive organisms, CO_2_-related mortality occurs during the earliest and thus smallest life stages (Dupont et al. 2008; Gobler et al. 2014), which rapidly decompose after death in experimental vessels. Hence, the requirement of individually sampling and genotyping all dying offspring may pose a challenge in species with smaller and more fragile offspring (e.g., mollusks, crustaceans) than newly hatched silverside larvae (∼5 mm SL, ∼2 mg wet weight; Baumann and Conover 2011b). In those cases, a potential solution might be to perform full factorial crosses and meticulously standardize the contribution of each cross to common rearing containers, which may then allow deducing the ‘dead’ from parental and survivor genotypes. In our case, meticulous siphoning resulted in near complete recovery (93%) of hatched individuals, and prior trials had ascertained that single larvae yield sufficient genomic material for amplification and genotyping (Malvezzi 2013). Whether this is feasible in other species has yet to be determined. We also assumed that a robust heritability estimate required approximately 1000 offspring and a good mixture of full siblings, half siblings, and unrelated individuals, which was feasible in this case by strip-spawning a large number of adults and using a large number of replicates (10 × 100 embryos). However, the costs associated with DNA extraction, amplification, and automated analysis of PCR products were approximately $10/individual (in 2013). Our approach was also time intensive, not the least because all experimental individuals (offspring + parents) had to be manually genotyped for each of nine polymorphic microsatellite loci via ABI-Analyzer scans [(total of 772 offspring + 71 parents)*9 loci = 7587 scans]. Adding to the time budget is the interpretation of genotypes and then evaluating intra- and inter-reader consistency.

In our final model (fixed + additive genetic random effect), the heritability estimate (±SE) for early life survival at high CO_2_ levels was 0.20 ± 0.07, which is well within the range of narrow sense heritability reported for survival in fish in the literature (Mousseau and Roff 1987; Law 2000). For example, heritability of early life survival in Atlantic salmon under aquaculture conditions ranged between 0 and 0.34 (Standal and Gjerde 1987; Jonasson 1993), while estimates for juvenile brook trout were 0.16–0.51 (Robison and Luempert 1984). In a meta-analysis of over 1100 narrow sense heritability estimates across taxa (Mousseau and Roff 1987) reported that 50% of the estimates for life history traits (e.g., survival, growth, fecundity) ranged between 0.14 and 0.34 (mean = 0.27 ± 0.03, *n* = 79), in contrast to generally higher heritability values associated with morphological traits like length or weight (mean = 0.51 ± 0.02, *n* = 140). The current understanding of the many sources of genetic variation in natural populations predicts this difference, because life history traits are more closely associated with fitness than more distantly related morphological traits (Falconer 1981; Mousseau and Roff 1987). Given the novelty of the approach and lack of heritability estimates for early life survival of metazoans under OA, the values are difficult to relate to previous works. However, heritability of early larval size under OA have been derived for a mussel and a sea urchin species (Sunday et al. 2011), where estimates at ambient CO_2_ levels (0.12 ± 0.09 and 0.14 ± 0.16, respectively) were higher than at elevated CO_2_ (0.00 ± 0.00 and 0.09 ± 0.10, respectively). Assuming that *h*^2^_size_ > *h*^2^_survival_, our study's heritability estimates for early survival in a fish (0.10–0.20) were notably above the two invertebrates studied thus far.

Does this mean that *M. menidia* will adapt relatively rapidly to OA? We believe that this conclusion is not warranted yet, given the current lack of understanding regarding the strength and form of OA-induced selection in the wild (but see Bednaršek et al. 2014). In addition to heritability, rapid evolutionary change requires strong, unidirectional selection. Conover and Munch (2002) found that strong artificial selection on *M. menidia* body size (only 10% of the largest/smallest adults were allowed to reproduce) resulted in a significant divergence between up- and down-selected lines within only four generations. Incidentally, the heritability of growth rate in this experiment was also 0.20. More recently, Brown et al. (2008) used this heritability estimate to model the rate of evolutionary change under more realistic selection differentials typical for commercial fisheries, reporting a comparable evolutionary change within 30 generations. OA-induced selection is surely weaker and not necessarily unidirectional. For example, exposure of newly fertilized embryos to 2300 μatm CO_2_ in the current experiment reduced average survival by 34%. In reality, however, high CO_2_ levels will not suddenly but gradually occur in the average open ocean over the next three centuries. In addition, indirect effects or other environmental changes (e.g., temperature, oxygen, plankton production) might mitigate or even reverse OA-induced selection (Pfister et al. 2014), which is inherently difficult to predict. Lastly, recent findings suggest that *M. menidia* offspring are CO_2_-sensitive only during some (early) parts of the spawning season, while becoming CO_2_ tolerant later in the season, when elevated respiration rates increase CO_2_ levels in tidal marsh spawning habitats (Murray et al. 2014; Baumann et al. 2015). Seasonally varying CO_2_ sensitivities are likely a result of parent–offspring linkages known as transgenerational plasticity, and its probable ubiquity adds yet another dimension to the question of species' evolutionary potential to OA (Salinas et al. 2013; Allan et al. 2014). One hypothesis is that coastal marine species such as *M. menidia* are adapted to highly variable CO_2_ conditions through intra- and transgenerational plasticity (Reusch 2013; Crozier and Hutchings 2014), which could effectively stall selection and evolutionary adaptation until perhaps reaching a threshold with adverse consequences (a form of evolutionary trap, Schlaepfer et al. 2002). In summary, we found that larval *M. menidia* are negatively affected by high CO_2_ levels and that early life survival has a significant additive genetic component that could elicit an evolutionary response if selection pressures in the wild resemble – at least in direction – the artificial conditions in the laboratory. While challenging, this constitutes one of the most important issues for future OA research to address (Merilä and Hendry 2014; Pfister et al. 2014; Sunday et al. 2014).

**Figure 5:**
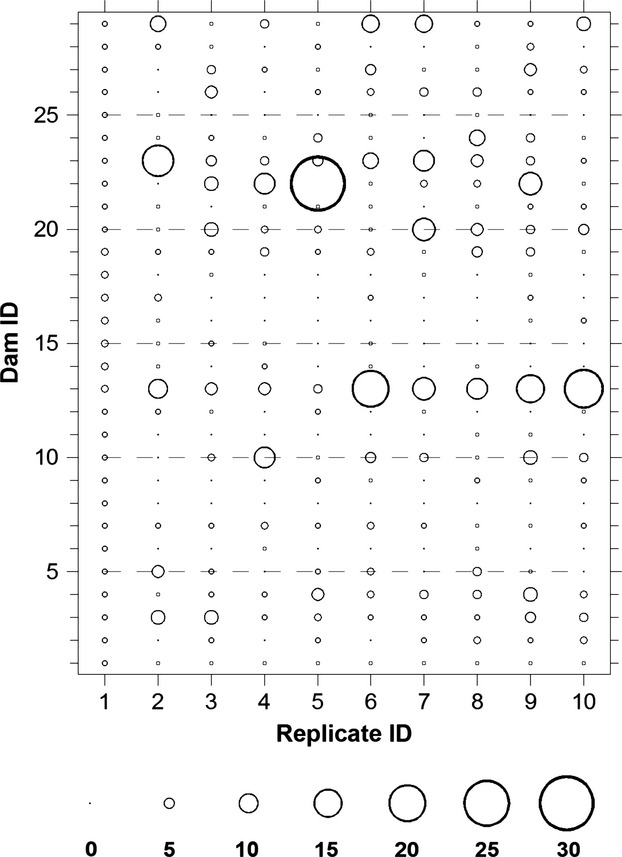
*Menidia menidia*. Distribution of offspring from 29 dams among 10 replicates based on the microsatellite based parent–offspring pedigree.

In addition to heritability, our quantitative genetic approach revealed links between phenotypic and genotypic differences among the 10 ‘replicates’ of this study. The strong statistical effect of replicate ID was most likely due to a nonrandom distribution of genotypes among experimental vessels, despite our meticulous efforts to randomize offspring at the beginning of the trial (Fig.5). Small biotic (e.g., larval density) and/or abiotic differences between replicates might have been contributing factors, even though daily temperature and pH monitoring suggested no systematic differences in physiochemical conditions. However, between-replicate survival varied threefold (28–76%, mean = 46%), while genotyping revealed that the number of dams (16–23, N_Parents_: 44–54), relative allelic richness (0.56–0.72), and observed heterozygosity (0.66–0.72) all varied substantially between replicates. The latter was significantly positively correlated to survival and explained almost 50% of the variation. All proxies for genetic variability were calculated across all individuals per replicate (regardless of dead and surviving), hence assuming that neutral (microsatellites) and adaptive genetic variability are correlated, our finding corroborates the notion that more genetic variability and a higher overall allelic richness among progeny positively affect survival (Ruzzante et al. 1996). This is consistent with studies showing diminished genetic variability in natural populations resulting in increased genetic bottlenecks, inbreeding, reductions in effective population size and the potential for reduced adaptability and productivity (Hauser et al. 2002; O'Leary et al. 2013).

Our work has emphasized the suitability of using a quantitative genetic approach to evaluate species evolutionary potential in the face of continuing OA. We believe that such estimates are of increasing interest to a research field that needs to address the questions of short- and long-term adaptability in marine organisms to allow a more realistic assessment of the OA threat.

## Data archiving statement

The microsatellite source data for this study were uploaded to Dryad (http://datadryad.org, doi: 10.5061/dryad.bd6vs).
